# Mortality of Philadelphia for January, February and March, 1856; Collated from the Health Office Record

**Published:** 1856-05

**Authors:** Wilson Jewell


*Mortality of Philadelphia for January, February and March, 1856; collated from the Health Office record.* By Wilson Jewell, M. D.

During the first quarter of the present year, beginning with Dec. 29th, 1855, and terminating with the 29th of March, 1856, a period of 91 days, the deaths recorded at the Health Office amounted to 2810, averaging nearly 31 deaths per day. An increase of 3½ per day, over those for the like period in 1855.

The deaths from diseases alone, number 2343, while the remainder were attributed to Still Born, External Causes, Old Age, Debility, Malformation and Unknown, amounting to 467. Of these, 103 were still born children.

The excess of deaths is with the males, amounting to 2.70 per cent. The excess of deaths among the males from external causes is equal to 45 per cent.

The deaths among children under five years amounted to 1190, equal to 45 per cent. of the whole number of deaths, exclusive of Still Born.

The highest mortality of any one disease was from Consumption of the lungs, 394. Small Pox, which has been very prevalent, furnished 238, and Scarlet Fever 189 deaths. The increase of deaths from these two forms of eruptive fevers, swelled the class of Zymotics to 670, equal to the deaths from the respiratory organs, which are always more prevalent during the winter season.

Under the head of Organs of Generation, we find recorded 26 deaths from Puerperal Fever, which is an unusually large number for one quarter. In 1855, the deaths from this disease for the entire year were only 41. It will be seen that we have departed from the usual classification, and placed the deaths from Puerperal Fever, with those charged to the organs of generation, as the most suitable place for them.

The deaths under twenty years, constitute 55 per cent. of the entire mortality, exclusive of Still Born. Those under one year, were nearly equal to one fourth of the whole.

The deaths fromConsumption of the Lungs, amounting to 394, or nearly 15 per cent, of the entire mortality, exclusive of Still Born, exceeded those for the like quarters since 1852

The increase of deaths, from "Disease of the Heart," (what kind ?) will be observed. This vague distinction constitutes nearly two-thirds of all the deaths from diseases of the organs of circulation, and shows the want of attention to diagnosis, in those affections involving the heart and its membranes.

Of the entire mortality for the quarter, there were 1106 in January ; 861 in February, and 843 in March. Of this total, 1443 were males, and 1367 females. The adults numbered 1176, while the children, or those under 20 years, amounted to 1634, of whom 849 were boys, and 785 girls, showing an excess of 3.91 per cent. of deaths among boys.

The deaths noted as from the Alms House were 182. Those of the colored population 138 ; from the country 35, and from the County Prison 2.

Small Pox, has been extensively prevalent during the entire quarter; the deaths averaging 18 per week. It assumed somewhat of an epidemic character; was not confined to any particular section of the city (the built portions.) It invaded all classes in the community. While each decennary period of life, from birth up to that between 50 and 60, contributed subjects for the mortuary record, infancy, or that term particularly between birth and 10 years, made up the heaviest amount.

Of the whole number for the quarter (238), one hundred and sixty-six, or 69.74 per cent., were in children under ten years of age.

The prevalence of Small Pox among children, and hence the large number of deaths, must be attributed in a great degree to a palpable defect in the ordinance for public vaccination, in which, for the two years past, the collectors of vaccine cases are dispensed with.

As an evidence of the correctness of our opinion as to the cause of this large mortality among the infantile population, we learn that the temporary collectors of vaccine cases appointed by the Board of Health for one month (March 1856), returned, during that period, the names of more than six thousand persons for vaccination! a large majority of whom were unvaccinated children.*

The ordinance alluded to has been amended, so as to provide for collectors of vaccine cases in the future.

At the present time the Small Pox is declining.

Scarlet Fever presents a large mortality for the quarter, averaging over 14 deaths for each week; children have been the principal sufferers, of whom 92; or nearly 49 per cent., were between the ages of 2 and 5.

No week passed without fresh cases of the disease. Many of these cases were unusually malignant in their character, while others were known only by the sequelæ.

Our long continued cold winter, remarkable for the amount of

* The results of vaccination in the Prussian army strongly exhibit the power we possess of controlling the ravages of Small Pox. (See Med. Exam. July 1855.) In Berlin, where vaccination is compulsory, not a single death occurred from Small Pox during the last year.—Ed.

snow that fell, and the quantity and thickness of the ice formed in the Delaware and Schuylkill Rivers, brought with it an increased prevalence of inflammatory diseases. Eruptive affections of every kind have also been unusually numerous. Erysipelas, carbuncles, boils, felons, together with eruptive fevers, have all appeared beyond the ordinary number during the quarter; while the acute diseases of the organs of respiration have been not only severe in their character but remarkably prevalent.

Puerperal fever, has also provided an uncommon share of the deaths for the quarter. The record says 26; but on reference to the deaths from diseases of the digestive organs, it will be found that 14 females died from peritoneal inflammation; some of whom, in all probability, were lying-in-women.

Fifteen of the above 26 deaths were from the Philadelphia Hospital, (Blockley,) where the disease has prevailed to a severe and fatal extent.

Dr. R. K. Smith, the chief resident physician of that institution, has furnished an interesting and valuable report of 27 cases, as they occurred in the lying-in ward, which will be found in the last (April) number of the Examiner.

The disease, however, has not been generally prevalent, and where it has existed it is now disappearing.

TABLE NO. 1.

*Deaths for the first quarter of 1856 classified.*

| | Jan. | Feb. | Mar. | Male. J. | Male. F. | Male. M. | Female. J. | Female. F. | Female. M. | Total. |
|---|---|---|---|---|---|---|---|---|---|---|
| 1 *Endemic & Contagious diseases* Zymotic or Epidemic | 292 | 204 | 174 | 158 | 108 | 93 | 134 | 96 | 81 | 670 |
| 2 *Uncertain or general seat,* Sporadic diseases | 150 | 113 | 112 | 68 | 57 | 68 | 82 | 56 | 44 | 375 |
| 3 Nervous system | 165 | 144 | 138 | 86 | 67 | 72 | 79 | 77 | 66 | 447 |
| 4 Organs of Respiration | 262 | 206 | 203 | 136 | 95 | 99 | 126 | 111 | 104 | 671 |
| 5 " Circulation | 35 | 24 | 25 | 19 | 10 | 14 | 16 | 14 | 11 | 84 |
| 6 Digestive organs | 43 | 41 | 38 | 26 | 22 | 18 | 17 | 19 | 20 | 122 |
| 7 Urinary " | 6 | 2 | 5 | 5 | 1 | 2 | 1 | 1 | 3 | 13 |
| 8 Organs of Generation | 16 | 11 | 11 | | 1 | | 16 | 10 | 11 | 38 |
| 9 " Locomotion | 5 | 4 | 7 | 2 | 3 | 6 | 3 | 1 | 1 | 16 |
| 10 Integumentary system | 7 | | 2 | 5 | | 1 | 2 | | 1 | 9 |
| 11 Old age | 20 | 18 | 22 | 6 | 9 | 5 | 14 | 9 | 17 | 60 |
| 12 External causes | 23 | 17 | 44 | 17 | 10 | 34 | 6 | 7 | 10 | 84 |
| Still Born | 61 | 53 | 49 | 35 | 28 | 27 | 26 | 25 | 22 | 163 |
| Unknown | 21 | 24 | 13 | 10 | 12 | 8 | 11 | 12 | 5 | 58 |
| | 1106 | 861 | 843 | 573 | 423 | 447 | 533 | 438 | 396 | 2810 |

### TABLE NO. 2.

### 1. *Endemic and Contagious Diseases—Zymotic or Epidemic.*

| | Male. | Female. | Under 1 yr. | 1 to 2. | 2 to 5. | 5 to 10. | 10 to 15. | 15 to 20. | 20 to 30. | 30 to 40. | 40 to 50. | 50 to 60. | 60 to 70. | 70 to 80. | 80 to 90. | 90 to 100. | 100 to 110. | Total. |
|---|---|---|---|---|---|---|---|---|---|---|---|---|---|---|---|---|---|---|
| Cholera | 1 | | | | | | | | | 1 | | | | | | | | 1 |
| " infantum | 1 | 1 | 1 | 1 | | | | | | | | | | | | | | 2 |
| " morbns | | 3 | | | | | | | | 1 | 2 | | | | | | | 3 |
| Croup | 46 | 39 | 20 | 14 | 44 | 6 | 1 | | | | | | | | | | | 85 |
| Diarrhœa | 5 | 3 | 2 | | | | | | | 1 | | 1 | 3 | | 1 | | | 8 |
| Dysentery | 6 | 4 | 3 | 1 | 2 | | | | 1 | 1 | | | 2 | | | | | 10 |
| Erysipelas | 19 | 10 | 11 | 3 | 1 | 1 | | 1 | 2 | 2 | 2 | 3 | 3 | | | | | 29 |
| Fever, | | 1 | 1 | | | | | | | | | | | | | | | 1 |
| " Bilious | | 3 | | | 1 | | | | | 1 | | | 1 | | | | | 3 |
| " Congestive | | 1 | | | | | | | | | 1 | | | | | | | 1 |
| " Nervous | 1 | 1 | | | | | 1 | | | | | | 1 | | | | | 2 |
| " Remittent | 2 | 2 | | | 1 | 1 | | | | | 1 | | | 1 | | | | 4 |
| " Scarlet | 98 | 91 | 19 | 32 | 92 | 32 | 3 | 3 | 5 | 3 | | | | | | | | 189 |
| " Typhoid | 21 | 18 | | 3 | 2 | 4 | 5 | 2 | 2 | 4 | 8 | 4 | 2 | 2 | 1 | | | 39 |
| " Typhus | 9 | 6 | | | | 2 | 1 | 1 | 5 | 1 | 2 | 3 | | | | | | 15 |
| Hooping Cough | 9 | 13 | 9 | 3 | 10 | | | | | | | | | | | | | 22 |
| Influenza | | 1 | | 1 | | | | | | | | | | | | | | 1 |
| Measles | 2 | 2 | 1 | 1 | 1 | 1 | | | | | | | | | | | | 4 |
| Small Pox | 132 | 106 | 49 | 28 | 60 | 29 | 10 | 9 | 30 | 10 | 4 | 8 | | 1 | | | | 238 |
| Syphilis | | 1 | | | | | | | 1 | | | | | | | | | 1 |
| Varicella | 2 | 1 | 3 | | | | | | | | | | | | | | | 3 |
| Varioloid | 5 | 4 | 2 | 4 | 1 | | | | 1 | | 1 | | | | | | | 9 |
| | 359 | 311 | 121 | 91 | 215 | 76 | 21 | 16 | 47 | 25 | 21 | 19 | 12 | 4 | 2 | | | 670 |

*2. Uncertain or General Seat—Sporadic Diseases.*

| | Male. | Female. | Under 1 yr. | 1 to 2. | 2 to 5. | 5 to 10. | 10 to 15. | 15 to 20. | 20 to 30. | 30 to 40. | 40 to 50. | 50 to 60. | 60 to 70. | 70 to 80. | 80 to 90. | 90 to 100. | 100 to 110. | Total. |
|---|---|---|---|---|---|---|---|---|---|---|---|---|---|---|---|---|---|---|
| Abscess | 10 | 8 | 5 | 1 | 2 | | | 2 | 2 | 2 | 1 | 2 | 1 | | | | | 18 |
| " Psoas | 1 | | | | | | | | | 1 | | | | | | | | 1 |
| Anasarca | 1 | 1 | 1 | | | | | | | 1 | | | | | | | | 2 |
| Cancer | 3 | 18 | | | | | | | | 2 | 2 | 13 | 2 | 2 | | | | 21 |
| Carcinoma | | 1 | | | | | | | | | 1 | | | | | | | 1 |
| Consumption of Throat | 1 | | | | | | | | | | | | | | | | | 1 |
| Cramp | 1 | 3 | 3 | | | 1 | | | 1 | | | | | | | | | 4 |
| Cyanosis | 13 | 5 | 17 | | | 1 | | | | | | | | | | | | 18 |
| Debility | 47 | 49 | 43 | 2 | 2 | | | | | 6 | 3 | 7 | 8 | 13 | 11 | 1 | | 96 |
| Disease of Neck | 1 | | 1 | | | | | | | | | | | | | | | 1 |
| Dropsy | 18 | 19 | 2 | 2 | 2 | 5 | 1 | | 3 | 2 | 5 | 5 | 5 | 3 | 2 | | | 37 |
| Gangrene | 4 | 2 | | | 1 | | | | 1 | 2 | | 1 | | | 1 | | | 6 |
| Gout | 1 | | | | | | | | | | | | 1 | | | | | 1 |
| Hemorrhage | 5 | 2 | 2 | | 2 | | | 1 | | 1 | 1 | | | | | | | 7 |
| Inanition | 31 | 19 | 44 | | | | | | | | | 3 | 1 | 2 | | | | 50 |
| Inflammation, Parotids | 1 | | | | | | | | | | | | | | 1 | | | 1 |
| " Throat | 3 | 2 | 1 | 1 | 1 | | | | 1 | | | | 1 | | | | | 5 |
| " Tonsils | | 1 | | 1 | | | | | | | | | | | | | | 1 |
| Malformation | 4 | 2 | 6 | | | | | | | | | | | | | | | 6 |
| Marasmus | 32 | 31 | 32 | 11 | 9 | | | 1 | 2 | 2 | 1 | 1 | 2 | 2 | | | | 63 |
| Mortification | 2 | 4 | | 2 | | | | | | | 1 | | 2 | | 1 | | | 6 |
| Osteo Sarcoma | | 1 | | | | | | | | | | | 1 | | | | | 1 |
| Quinsy | | 1 | | | | | | | | | | | | | 1 | | | 1 |
| Scirrhus | 1 | | | | | | | | | 1 | | | | | | | | 1 |
| Sore Throat | | 2 | | | 2 | | | | | | | | | | | | | 2 |
| Scrofula | 7 | 2 | 2 | 1 | 2 | 1 | | | | | | 3 | | | | | | 9 |
| Tabes Mesenterica | 3 | 4 | 3 | 2 | 2 | | | | | | | | | | | | | 7 |
| Tumour | 1 | | | | | | | | | | | 1 | | | | | | 1 |
| Ulceration | | 2 | | 1 | 1 | | | | | | | | | | | | | 2 |
| " Mouth | | 1 | | 1 | | | | | | | | | | | | | | 1 |
| " Throat | 2 | 2 | 2 | | | 1 | | 1 | | | | | | | | | | 4 |
| | 193 | 182 | 164 | 25 | 26 | 9 | 1 | 5 | 10 | 20 | 15 | 36 | 24 | 22 | 17 | 1 | | 375 |

*3. Nervous Diseases.*

| | Male. | Female. | Under 1 yr. | 1 to 2. | 2 to 5. | 5 to 10. | 10 to 15. | 15 to 20. | 20 to 30. | 30 to 40. | 40 to 50. | 50 to 60. | 60 to 70. | 70 to 80. | 80 to 90. | 90 to 100. | 100 to 110. | Total. |
|---|---|---|---|---|---|---|---|---|---|---|---|---|---|---|---|---|---|---|
| Apoplexy | 16 | 12 | | | | | | | 1 | 5 | 5 | 7 | 6 | 2 | 2 | | | 28 |
| Congestion of Brain | 20 | 12 | 8 | 4 | 4 | 2 | 2 | 1 | 1 | 4 | 1 | 3 | | 1 | 1 | | | 32 |
| Convulsions | 66 | 74 | 85 | 16 | 22 | 7 | 4 | | 2 | | 1 | 2 | | 1 | | | | 140 |
| Disease of Brain | 15 | 11 | 6 | | 5 | 4 | 1 | | | | 3 | 2 | 1 | 3 | 1 | | | 26 |
| Dropsy " | 30 | 29 | 24 | 16 | 12 | 4 | | | 2 | | 1 | | | | | | | 59 |
| Effusion " | 11 | 10 | 9 | 4 | 5 | 1 | | | | 1 | | | 1 | | | | | 21 |
| Epilepsy | 3 | 6 | | 2 | | | | | 1 | 2 | 1 | 2 | 1 | | | | | 9 |
| Hysteria | | 3 | | | | | | | 1 | 1 | 1 | | | | | | | 3 |
| Inflammation of Brain | 39 | 38 | 25 | 13 | 14 | 8 | 3 | 5 | 3 | 3 | 2 | 1 | | | | | | 77 |
| Mania | 1 | 3 | | | | | | | | 3 | | 1 | | | | | | 4 |
| " a Potu | 6 | 3 | | | | | | | 2 | 4 | 2 | 1 | | | | | | 9 |
| Palsy | 13 | 18 | | | 1 | | | 1 | 3 | 3 | 3 | 1 | 7 | 7 | 3 | 1 | 1 | 31 |
| Softening of Brain | 4 | 3 | 1 | | | | | | 1 | 2 | | 1 | 1 | 1 | | | | 7 |
| Tetanus | 1 | | | | | | | | 1 | | | | | | | | | 1 |
| | 225 | 222 | 158 | 55 | 63 | 26 | 10 | 7 | 18 | 28 | 20 | 21 | 17 | 15 | 7 | 1 | 1 | 447 |

4. *Organs of Respiration.*

| | Male. | Female. | Under 1 yr. | 1 to 2. | 2 to 5. | 5 to 10. | 10 to 15. | 15 to 20. | 20 to 30. | 30 to 40. | 40 to 50. | 50 to 60. | 60 to 70. | 70 to 80. | 80 to 90. | 90 to 100. | 100 to 110. | Total. |
|---|---|---|---|---|---|---|---|---|---|---|---|---|---|---|---|---|---|---|
| Asthma | 5 | 1 | 3 | 1 | 1 | | | | | | | | | | | 1 | | 6 |
| Apnœa | 1 | | | | | | | | | | | | | 1 | | | | 1 |
| Congestion of Lungs | 8 | 9 | 5 | 2 | 4 | 1 | | 1 | | 1 | | 2 | 1 | | | | | 17 |
| Consumption " | 182 | 212 | 7 | 3 | 9 | 6 | 8 | 37 | 142 | 83 | 50 | 27 | 15 | 4 | 3 | | | 394 |
| Disease of " | 7 | 1 | 3 | 1 | | | | | | 2 | 2 | | | | | | | 8 |
| " Chest | 2 | | | | | | | | 1 | | | | | 1 | | | | 2 |
| Dropsy of " | 10 | 6 | | | 1 | 5 | 1 | | | 4 | 1 | | 1 | 1 | 1 | 1 | | 16 |
| Empyema | | 1 | | | | | | | 1 | | | | | | | | | 1 |
| Emphysema | | 1 | | | | | | | 1 | | | | | | | | | 1 |
| Effusion on Chest | | 1 | | | | | | | | | | | 1 | | | | | 1 |
| Hemorrhage of Lungs | 1 | 4 | | | | | | 1 | 2 | 1 | 1 | | | | | | | 5 |
| Inflamm'n of Bronchi | 45 | 45 | 40 | 12 | 14 | | 2 | | 3 | 1 | 2 | 6 | 4 | 4 | 2 | | | 90 |
| " Chest | 2 | 1 | 1 | | | | | | | | 1 | 1 | | | | | | 3 |
| " Larynx | 9 | 2 | 2 | 2 | 2 | 1 | | | 1 | | 1 | | 2 | | | | | 11 |
| " Lungs | 56 | 55 | 32 | 11 | 14 | | 3 | 6 | 12 | 9 | 8 | 6 | 7 | 1 | 1 | 1 | | 111 |
| " Pleura | | 2 | 1 | | | | | | | | | | 1 | | | | | 2 |
| " Trachea | 1 | | | | 1 | | | | | | | | | | | | | 1 |
| Ulceration of Larynx | 1 | | | | 1 | | | | | | | | | | | | | 1 |
| | 330 | 341 | 94 | 32 | 47 | 13 | 14 | 45 | 163 | 101 | 66 | 42 | 32 | 12 | 7 | 3 | | 671 |

5. *Organs of Circulation.*

| | Male. | Female. | Under 1 yr. | 1 to 2. | 2 to 5. | 5 to 10. | 10 to 15. | 15 to 20. | 20 to 30. | 30 to 40. | 40 to 50. | 50 to 60. | 60 to 70. | 70 to 80. | 80 to 90. | 90 to 100. | 100 to 110. | Total. |
|---|---|---|---|---|---|---|---|---|---|---|---|---|---|---|---|---|---|---|
| Anæmia | 2 | 1 | 1 | 1 | | 1 | | | | | | | | | | | | 3 |
| Aneurism | 2 | | | | | | | | | 1 | 1 | | | | | | | 2 |
| Angina Pectoris | | 1 | | | | | | | | 1 | | | | | | | | 1 |
| Disease of Heart | 35 | 31 | 1 | 2 | 3 | 3 | 2 | 2 | 5 | 8 | 11 | 11 | 11 | 5 | | | | 66 |
| Dropsy " | 1 | 3 | | | 1 | | | | | | | 1 | 2 | | 2 | | | 4 |
| Enlargem't " | 3 | 2 | | 1 | 1 | | 1 | | | 1 | | | 1 | | | | | 5 |
| Pyohæmia | | 1 | | | | | | | | | 1 | | | | | | | 1 |
| Syncope | | 1 | | | | | | | | | | | | 1 | | | | 1 |
| Rupture of Blood-vessel | | 1 | | | | 1 | | | | | | | | | | | | 1 |
| | 43 | 41 | 2 | 4 | 5 | 5 | 3 | 2 | 5 | 11 | 13 | 12 | 14 | 6 | 2 | | | 84 |

### 6. *Digestive Organs.*

| | Male. | Female. | Under 1 yr. | 1 to 2. | 2 to 5. | 5 to 10. | 10 to 15. | 15 to 20. | 20 to 30. | 30 to 40. | 40 to 50. | 50 to 60. | 60 to 70. | 70 to 80. | 80 to 90. | 90 to 100. | 100 to 110. | Total. |
|---|---|---|---|---|---|---|---|---|---|---|---|---|---|---|---|---|---|---|
| Aphthæ | | 1 | 1 | | | | | | | | | | | | | | | 1 |
| Carcinoma of Stomach | 1 | | | | | | | | | | | 1 | | | | | | 1 |
| Cirrhosis | 1 | | | | | | | | | | | | 1 | | | | | 1 |
| " of Liver | 1 | | | | | | | | | | | 1 | | | | | | 1 |
| Cancrum Oris | 2 | 1 | | | 2 | | | | | 1 | | | | | | | | 3 |
| Congestion of Bowels | 1 | | | | | | | 1 | | | | | | | | | | 1 |
| Colic | 3 | 1 | 2 | | | | | | | 1 | | | | 1 | | | | 4 |
| Constipation | | 2 | 1 | | | | | | | | | | 1 | | | | | 2 |
| Consumption of Bowels | 1 | 1 | 1 | | | | | | 1 | | | | | | | | | 2 |
| Disease of Abdomen | 1 | | 1 | | | | | | | | | | | | | | | 1 |
| " Liver | 3 | | | | | | | | | 1 | 1 | 1 | | | | | | 3 |
| " Stom. & Bowels | 2 | 4 | 1 | | 2 | | | | | | | 1 | 1 | | 1 | | | 6 |
| Dropsy of Abdomen | 1 | 1 | | | | | | | | 2 | | | | | | | | 2 |
| Hemorrhage of Umbilicus | | 1 | 1 | | | | | | | | | | | | | | | 1 |
| Hernia | 1 | 1 | 1 | | | | | | | | | | 1 | | | | | 2 |
| Hardening of Liver | 1 | | | | | | | | | | 1 | | | | | | | 1 |
| Inflammat. Stom. & Bowels | 24 | 19 | 7 | 4 | 3 | 3 | 2 | 3 | 6 | 4 | 4 | 3 | 3 | 1 | | | | 43 |
| " Liver | 9 | 1 | | | 3 | 1 | 1 | | | 2 | 2 | 1 | | | | | | 10 |
| " Peritoneum | 5 | 14 | | | 2 | | | 3 | 8 | 5 | | | 1 | | | | | 19 |
| " Spleen | | 1 | | | | | | | 1 | | | | | | | | | 1 |
| Intussusception | 1 | 1 | | | | | 1 | | | 1 | | | | | | | | 2 |
| Jaundice | 1 | | | 1 | | | | | | | | | | | | | | 1 |
| Obstruction of Bowels | 4 | 1 | 1 | 1 | | | | | | 1 | | | 1 | 1 | | | | 5 |
| Softening of Stomach | 1 | 1 | | | | | | | | | | 1 | 1 | | | | | 2 |
| Sore Mouth | | 1 | | | 1 | | | | | | | | | | | | | 1 |
| Teething | 1 | 3 | 4 | | | | | | | | | | | | | | | 4 |
| Ulceration of Bowels | | 1 | | | | | | | | | | | 1 | | | | | 1 |
| Worms | 1 | | 1 | | | | | | | | | | | | | | | 1 |
| | 66 | 56 | 22 | 6 | 13 | 4 | 4 | 7 | 16 | 18 | 8 | 9 | 11 | 3 | 1 | | | 122 |

### 7. *Urinary Organs.*

| | Male. | Female. | Under 1 yr. | 1 to 2. | 2 to 5. | 5 to 10. | 10 to 15. | 15 to 20. | 20 to 30. | 30 to 40. | 40 to 50. | 50 to 60. | 60 to 70. | 70 to 80. | 80 to 90. | 90 to 100. | 100 to 110. | Total. |
|---|---|---|---|---|---|---|---|---|---|---|---|---|---|---|---|---|---|---|
| Diabetes | 4 | | | | | | | 1 | | | | 1 | 1 | 1 | | | | 4 |
| Disease of Kidneys | 3 | 1 | | | | | | | 1 | 2 | | | | | 1 | | | 4 |
| Hæmaturia | | 1 | | | | | | | 1 | | | | | | | | | 1 |
| Inflammation of Kidneys | 1 | 1 | | | 1 | | | | 1 | | | | | | | | | 2 |
| " Bladder | | 2 | | | | | | | | | 1 | | | | 1 | | | 2 |
| | 8 | 5 | | | 1 | | | 1 | 3 | 2 | 1 | 1 | 1 | 1 | 2 | | | 13 |

8. *Organs of Generation.*

| | Male. | Female. | Under 1 yr. | 1 to 2. | 2 to 5. | 5 to 10. | 10 to 15. | 15 to 20. | 20 to 30. | 30 to 40. | 40 to 50. | 50 to 60. | 60 to 70. | 70 to 80. | 80 to 90. | 90 to 100. | 100 to 110. | Total. |
|---|---|---|---|---|---|---|---|---|---|---|---|---|---|---|---|---|---|---|
| Amenorrhœa | | 1 | | | | | | | 1 | | | | | | | | | 1 |
| Cancer of Uterus | | 1 | | | | | | | | | | | 1 | | | | | 1 |
| Disease of Prostate Gland | 1 | | | | | | | | | | | | | | 1 | | | 1 |
| " Uterus | | 3 | | | | | | | 1 | 1 | 1 | | | | | | | 3 |
| Fever, Puerperal | | 26 | | | | | | 1 | 18 | 5 | 2 | | | | | | | 26 |
| Hemorrhage " | | 1 | | | | | | | | 1 | | | | | | | | 1 |
| " Uterus | | 3 | | | | | | | | 1 | 1 | | | | 1 | | | 3 |
| Inflammation of Ovaries | | 1 | | | | | | | | 1 | | | | | | | | 1 |
| " Uterus | | 1 | | | | | | | | 1 | | | | | | | | 1 |
| | 1 | 37 | | | | | | 1 | 20 | 10 | 4 | | 1 | | 2 | | | 38 |

9. *Organs of Locomotion.*

| | Male. | Female. | Under 1 yr. | 1 to 2. | 2 to 5. | 5 to 10. | 10 to 15. | 15 to 20. | 20 to 30. | 30 to 40. | 40 to 50. | 50 to 60. | 60 to 70. | 70 to 80. | 80 to 90. | 90 to 100. | 100 to 110. | Total. |
|---|---|---|---|---|---|---|---|---|---|---|---|---|---|---|---|---|---|---|
| Caries of Spine | 1 | | | | | 1 | | | | | | | | | | | | 1 |
| Disease " | 3 | 2 | | 1 | | | | 1 | 1 | | 1 | 1 | | | | | | 5 |
| " of Hip | 1 | | | | | | | 1 | | | | | | | | | | 1 |
| Necrosis | 1 | | | | | | | | | | | | | 1 | | | | 1 |
| Rheumatism | 3 | 3 | | | 1 | | 1 | | 1 | | 1 | 1 | | | 1 | | | 6 |
| Softening of Spinal Marrow | 1 | | | | | | | | | | | | 1 | | | | | 1 |
| Spina Bifida | 1 | | 1 | | | | | | | | | | | | | | | 1 |
| | 11 | 5 | 1 | 1 | 1 | 1 | 1 | 2 | 2 | | 2 | 2 | 1 | 1 | 1 | | | 16 |

10. *Integumentary System.*

| | Male. | Female. | Under 1 yr. | 1 to 2. | 2 to 5. | 5 to 10. | 10 to 15. | 15 to 20. | 20 to 30. | 30 to 40. | 40 to 50. | 50 to 60. | 60 to 70. | 70 to 80. | 80 to 90. | 90 to 100. | 100 to 110. | Total. |
|---|---|---|---|---|---|---|---|---|---|---|---|---|---|---|---|---|---|---|
| Anthrax | 2 | | | | | | | | 1 | | | 1 | | | | | | 2 |
| Disease of Skin | | 1 | 1 | | | | | | | | | | | | | | | 1 |
| Fistula | 1 | | | | | | | | | 1 | | | | | | | | 1 |
| Lepra | | 1 | | | | | | | | | | | | 1 | | | | 1 |
| Purpura Hemorrhagica | 3 | 1 | 2 | | | | | | 1 | 1 | | | | | | | | 4 |
| | 6 | 3 | 3 | | | | | | 2 | 2 | | 1 | | 1 | | | | 9 |

11. *Old Age.*

| | Male. | Female. | Under 1 yr. | 1 to 2. | 2 to 5. | 5 to 10. | 10 to 15. | 15 to 20. | 20 to 30. | 30 to 40. | 40 to 50. | 50 to 60. | 60 to 70. | 70 to 80. | 80 to 90. | 90 to 100. | 100 to 110. | Total. |
|---|---|---|---|---|---|---|---|---|---|---|---|---|---|---|---|---|---|---|
| Old Age | 20 | 40 | | | | | | | | | | | 4 | 16 | 27 | 13 | | 60 |

12. *External Causes.*

| | Male. | Female. | Under 1 yr. | 1 to 2. | 2 to 5. | 5 to 10. | 10 to 15. | 15 to 20. | 20 to 30. | 30 to 40. | 40 to 50. | 50 to 60. | 60 to 70. | 70 to 80. | 80 to 90. | 90 to 100. | 100 to 110. | Total. |
|---|---|---|---|---|---|---|---|---|---|---|---|---|---|---|---|---|---|---|
| Asphyxia | 1 | | | | | | | | 1 | | | | | | | | | 1 |
| Burns | 6 | 7 | | 3 | 4 | | | | 2 | 3 | | 1 | | | | | | 13 |
| Casualties | 29 | 4 | 1 | | 1 | | | | 12 | 10 | 4 | 2 | | | | | | 33 |
| Drowned | 4 | 2 | | | | | 2 | 1 | 1 | 3 | 2 | | | | | | | 6 |
| Exhaustion | 2 | 2 | | | | | | | 2 | 2 | | | | | | | | 4 |
| Exposure | 7 | 1 | 1 | | | | | | | 4 | 2 | 1 | | | | | | 8 |
| Fracture | 2 | | | | | | | | 1 | | | | 1 | | | | | 2 |
| Intemperance | 6 | 2 | | | | | | | | 3 | 3 | 2 | | | | | | 8 |
| Poisoning | | 2 | | | | | | | | 1 | | | 1 | | | | | 2 |
| Suffocation | 1 | 2 | | 1 | 1 | | | | | | | 1 | | | | | | 3 |
| Suicide | 2 | 1 | | | | | | | 2 | | 1 | | | | | | | 3 |
| Wound | 1 | | | | | | | | | | | | 1 | | | | | 1 |
| | 61 | 23 | 2 | 4 | 6 | | 2 | 1 | 21 | 26 | 12 | 7 | 3 | | | | | 84 |
| Still Born | 90 | 73 | 163 | | | | | | | | | | | | | | | 163 |
| Unknown | 30 | 28 | 24 | 1 | 3 | 3 | 1 | | 4 | 4 | 7 | 6 | 2 | 3 | | | | 58 |

TABLE NO. 3.

*Deaths for the First Quarter of 1856, at fifteen distinct periods of life*

| | |
|---|---|
| Under 1 year, | 591 |
| 1 to 2 | 219 |
| 2 to 5 | 380 |
| 5 to 10 | 137 |
| 10 to 15 | 57 |
| 15 to 20 | 87 |
| 20 to 30 | 311 |
| 30 to 40 | 247 |
| 40 to 50 | 169 |
| 50 to 60 | 156 |
| 60 to 70 | 122 |
| 70 to 80 | 84 |
| 80 to 90 | 68 |
| 90 to 100 | 18 |
| 100 to 110 | 1 |
| | 2647 |
| Still Born | 163 |
| Total, | 2810 |

Included within the above table were 182 from the Blockley Alms House; 138 Blacks; 2 from the County Prison, and 35 from the country, as follows:—

| | Jan. | Feb. | March. | Total. |
|---|---|---|---|---|
| Almshouse, | 73 | 49 | 60 | 182 |
| Blacks, | 58 | 39 | 41 | 138 |
| County Prison, | | | 2 | 2 |
| Country, | 9 | 4 | 22 | 35 |
| | 140 | 92 | 125 | 357 |

